# Socioeconomic Differences in Lifetime and Past 30-Day E-Cigarette, Cigarette, and Dual Use: A State-Level Analysis of Utah Youth

**DOI:** 10.3390/ijerph19137557

**Published:** 2022-06-21

**Authors:** Christopher Cambron, Kaitlyn J. Thackeray

**Affiliations:** College of Social Work, University of Utah, Salt Lake City, UT 84112, USA; kaitlyn.thackeray@hsc.utah.edu

**Keywords:** e-cigarettes, cigarettes, neighborhood poverty, youth substance use, health disparities

## Abstract

Socioeconomic disparities in combustible cigarette use are well established among youth in the United States and lead to substantial health effects. Given the noteworthy rise in electronic cigarette (e-cigarette) use among youth in recent years, health professionals have expressed concern that e-cigarette use will follow similar socioeconomic patterns. The current study examined this question using a 2019 state-representative sample of youth in grades 6, 8, 10, and 12 from Utah (*N* = 78,740). Logistic regression models estimated associations between neighborhood- and individual-level factors with lifetime and past 30-day e-cigarette, combustible cigarette, and dual use across 267 neighborhoods. After controlling for individual-level sociodemographic factors, results indicated that youth living in higher-poverty neighborhoods were at a significantly increased risk of lifetime e-cigarette, combustible cigarette, and dual use. Additionally, youth living in households with higher levels of education were at a significantly lower risk of lifetime and past 30-day e-cigarette, cigarette, and dual use. Results suggest that e-cigarettes may follow a similar pattern of socioeconomic disparities among youth as combustible cigarettes. Additionally, most youth using combustible cigarettes also used e-cigarettes, suggesting that any potential harms from e-cigarettes may exacerbate existing socioeconomic disparities in health effects from combustible cigarette use. Research should continue to examine individual- and neighborhood-level socioeconomic disparities in youth e-cigarette, combustible cigarette, and dual use.

## 1. Introduction

Across the United States, rates of combustible cigarette use among youth and adults have dropped consistently throughout recent decades [1,2]. However, the health benefits from reductions in cigarette smoking have not been equally distributed across the population [3]. While population-level cigarette use has dropped, lower socioeconomic status (SES) groups have continued to smoke cigarettes at higher rates, leading to widening socioeconomic-related health disparities [4]. Electronic cigarettes (e-cigarettes) were introduced in the United States in 2007 in part to provide a path for longtime cigarette smokers to transition away from tobacco [2]. In recent years, e-cigarettes have become increasingly popular among youth. By 2014, the rapid increase in e-cigarette uptake among youth prompted the Surgeon General to declare an epidemic of e-cigarette use among youth [5]. While it is generally believed that e-cigarettes are less harmful than combustible cigarettes, the recency of the e-cigarette epidemic among youth makes it difficult to assess long-term health effects [6]. There remains substantial concern that early exposure to nicotine, heavy metals, and other harmful chemicals can have lasting health effects [5]. Additionally, there is growing evidence to suggest that e-cigarette use among youth increases the likelihood of subsequent combustible cigarette use [4,7].

Health professionals have expressed concern that e-cigarette use will follow a similar socioeconomic path as combustible cigarettes among youth [8]. If so, lower-SES youth exposed to both combustible cigarettes and e-cigarettes may experience compounding health effects that further exacerbate socioeconomic disparities in health across a lifetime [3]. To date, studies examining individual- and family-level socioeconomic differences in e-cigarette use have reported either no differences in e-cigarette use by SES or greater use among more affluent groups [9,10]. Importantly, these studies have not examined the socioeconomic features of social contexts in which youth live. Youth development researchers have long recognized that health behaviors are strongly influenced by the socioeconomic contexts in which these behaviors take place [11,12,13,14]. Therefore, it is important to examine the socioeconomic features of the schools and neighborhoods in which health behaviors take shape among youth [15]. To date, only two studies could be located examining this question for youth e-cigarette use. First, Shih and colleagues tested neighborhood SES differences in the use of multiple substances including e-cigarettes in a community sample of California youth [16]. Significant differences by neighborhood SES were not observed for the use of any substance among these youth. Second, Springer and colleagues reported that sixth-grade students attending lower-SES schools in Texas had more favorable attitudes toward e-cigarette use compared with comparable youth attending higher-SES schools [17]. These results suggest that youth attending lower-SES schools might be more susceptible to future e-cigarette use [17].

The primary goal of this study is to examine the socioeconomic differences in e-cigarette, cigarette, and dual e-cigarette/cigarette use. Comparing neighborhood- and individual-level socioeconomic differences across e-cigarette, cigarette, and dual users can provide novel information on the potential for e-cigarettes to follow a similar socioeconomic path as combustible cigarette use [8]. To expand on the limited research examining socioeconomic contexts for e-cigarette among youth, the current study tested six interrelated hypotheses using a 2019 state-representative sample from the United States. Statistical models estimated differences in the likelihood of lifetime and past 30-day e-cigarette, cigarette, and dual use by neighborhood- and individual-level socioeconomic factors after accounting for individual-level sociodemographic factors. Given the well-documented associations between SES and cigarette smoking paired with recent evidence of increased susceptibility to e-cigarettes in lower-SES schools [4,11,13,17], we hypothesized that youth living in lower-SES neighborhoods would be more likely to report e-cigarette, cigarette, and dual use.

## 2. Materials and Methods

### 2.1. Sample

Data for this study were drawn from the 2019 Prevention Needs Assessment (PNA) survey. This survey is administered every 2 years to youth by the Utah Division of Substance Abuse and Mental Health [18]. The PNA combines elements of commonly administered surveys of youth behavior including the CDC’s Youth Behavioral Risk Factor Surveillance System (YRBSS) and the Communities that Care (CTC) Youth Survey [19,20]. The PNA employs a complex sampling design whereby data are stratified by school district, clustered by grade, and weighted to approximate statewide demographics of youth. Parents or guardians of respondents provided active consent for participation. Validated and anonymized data from students in grades 6, 8, 10, and 12 were used for the current study (*N* = 86,364).

### 2.2. Measures

#### 2.2.1. Lifetime and Past 30-Day E-Cigarette, Cigarette, and Dual Use

Lifetime e-cigarette use was measured by a single item, “Have you ever tried vape products such as e-cigarettes, vape pens, or mods?” with response options coded as 0 = no and 1 = yes. Past 30-day e-cigarette use was measured by a single item, “During the past 30 days, on how many days did you use vape products such as e-cigarettes, vape pens, or mods?” with seven response options (0 day, 1 or 2 days, 3 to 5 days, 6 to 9 days, 10 to 19 days, 20 to 29 days, all 30 days). Lifetime and past 30-day cigarette use were measured with identically structured questions that used phrasing for cigarettes as opposed to e-cigarettes. Given the small cell sizes for many youth, categories indicating any e-cigarette use were collapsed such that 0 = no use and 1 = any use. Youth missing on past 30-day e-cigarette and cigarette use and reporting no lifetime use were coded as 0. Dual e-cigarette and cigarette use measures were constructed for those who had non-missing data for both e-cigarette and cigarette use.

#### 2.2.2. Neighborhood Characteristics

Neighborhood poverty was measured by 5-year estimates from the American Community Survey (ACS) [4]. Estimates of the percentages of families below the poverty line, individuals receiving public assistance, individuals 25 or older without high school diploma, and individuals unemployed and in the workforce were gathered from 278 available ZIP code tabulation areas (ZCTAs) across Utah via the *tidycensus* package in R v4.1 [21]. Similar to previous studies, a measure of neighborhood poverty was extracted from principal components analysis (PCA) summarizing ACS variables [22]. Across all ZCTAs in the state, neighborhood poverty principal component scores ranged from −2.07 to 6.48 (M = −0.29, SD = 1.42). Urban and rural designations for each ZCTA were drawn from rural–urban community area (RUCA) codes for 2010 [7]. RUCA codes for metro- and micropolitan areas were classified as urban, while codes for small-town and rural areas were coded as rural [23].

#### 2.2.3. Demographics

Age, gender, race/ethnicity, and household education were each measured by a single item. Gender provided the response options woman/girl, man/boy, transgender, and other. Responses of transgender and other were set to missing given an insufficient sample size. Race/ethnicity provided the response options American Indian or Alaskan Native, Asian, Black or African American, Hispanic/Latino, Native Hawaiian or other Pacific Islander, and White. Race/ethnicity categories were not mutually exclusive and were collapsed into two binary variables for non-White and Hispanic/Latino. Youth were asked to report the highest level of schooling completed by an adult in their household. Responses were coded as high school or less, some college, college degree, and graduate degree.

### 2.3. Analytic Plan

Data were analyzed with Mplus v8.7 [8]. Logistic regression models for lifetime and past 30-day e-cigarette, cigarette, and dual use were clustered by ZCTA, stratified by school district, and weighted to approximate population characteristics. Neighborhood poverty and age were standardized with a mean of 0 and a standard deviation of 1 prior to analysis. Odds ratios (ORs) and 95% confidence intervals (CI) were computed from logistic regression estimates. Cases with nonexistent or missing ZCTA codes could not be included in the statistical models, leaving an analytic sample of 78,740. Data were present for 96.3% of possible data points (1,061,915 out of a possible 1,102,360), and missing data were handled by full information maximum likelihood. The PCA was conducted using the *psych* package in R v4.1 [9]. Multiple sensitivity tests were conducted to examine the robustness of the results. Multiple group models examined grade-specific effects and produced substantively similar results in most cases but did not converge for all estimates. Unweighted multilevel models for all grades combined were examined and produced substantively identical results to those presented below. Table 1 provides descriptive statistics for the sample. Table 2 provides the results of the PCA for neighborhood poverty.

## 3. Results

Results of six logistic regression model examining neighborhood- and individual-level differences in youth lifetime and past 30-day e-cigarette, cigarette, and dual use are described below. For neighborhood-level factors, youth living in higher-poverty neighborhoods were significantly more likely to report lifetime e-cigarette, cigarette, and dual use. Significant differences between rural and urban neighborhoods were not observed after accounting for poverty levels. For individual-level factors, as household education levels increased, youth were significantly less likely to report lifetime and past 30-day e-cigarette, cigarette, and dual use. As expected, older youth were significantly more likely to report lifetime and past 30-day e-cigarette, cigarette, and dual use. Male youth were significantly more likely to report lifetime e-cigarette, cigarette, and dual use, but these trends did not hold for past 30-day use. Specific to e-cigarettes, non-White youth and Hispanic/Latino youth were significantly more likely to report lifetime use, and non-White youth were significantly more likely to report past 30-day e-cigarette use. For both cigarette and dual use, Hispanic/Latino youth were significantly more likely to report lifetime use, and non-White youth were significantly less likely to report past 30-day use. Models explained 24% and 19% of the variance in lifetime and past 30-day e-cigarette use, respectively; 20% and 23% of the variance in lifetime and past 30-day cigarette use, respectively; and 29% and 29% of the variance in lifetime and past 30-day dual use, respectively.

Interpretation of adjusted ORs for SES differences indicated that youth living in higher-poverty neighborhoods were at greater odds for lifetime e-cigarette, cigarette, and dual use. For each one standard deviation unit increase in neighborhood poverty PCA scores, odds of reporting lifetime e-cigarette, cigarette, and dual use increased by a factor of 1.12 (95% CI: 1.06, 1.19), 1.14 (95% CI: 1.08, 1.21), and 1.16 (95% CI: 1.08, 1.24), respectively, after accounting for other neighborhood- and individual-level sociodemographic factors. Additionally, youth living in households with higher levels of education were at reduced odds for both lifetime and past 30-day e-cigarette, cigarette, and dual use. For each one unit increase in household education, odds of reporting lifetime e-cigarette, cigarette, and dual use decreased by a factor of 0.60 (95% CI: 0.57, 0.62), 0.57 (95% CI: 0.54, 0.61), and 0.50 (95% CI: 0.46, 0.53), respectively, after accounting for neighborhood factors and other individual-level sociodemographic factors. For each one unit increase in household education, odds of reporting past 30-day e-cigarette, cigarette, and dual use also decreased by a factor of 0.61 (95% CI: 0.57, 0.64), 0.51 (95% CI: 0.44, 0.59), and 0.44 (95% CI: 0.37, 0.52), respectively, after accounting for neighborhood factors and other individual-level sociodemographic factors. 

**Figure 1:**
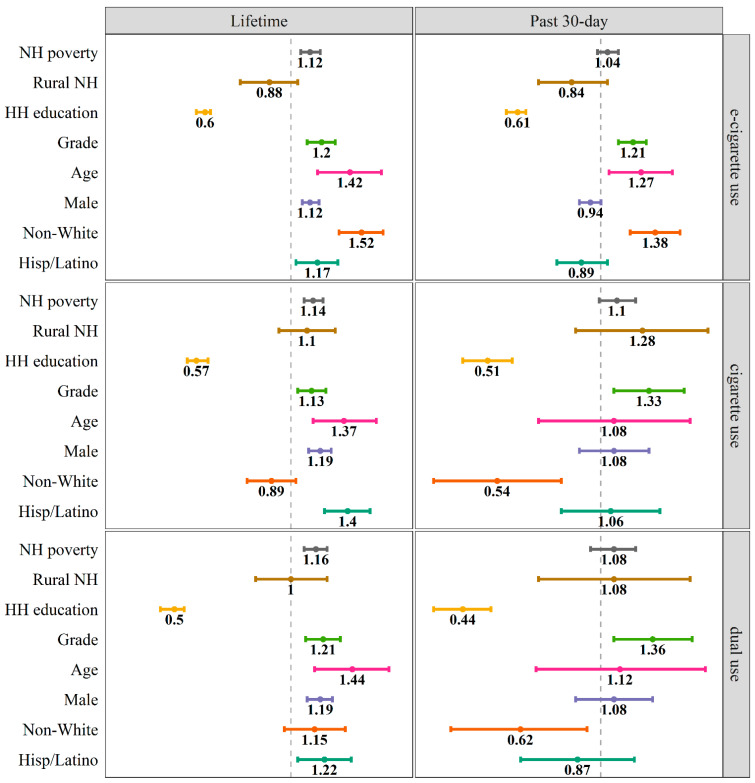
Adjusted odds ratios and 95% confidence intervals from logistic regression models for neighborhood- and individual-level associations with lifetime and past 30-day e-cigarette, cigarette, and dual use. NH = neighborhood and HH = household. NH poverty and age were standardized prior to modeling. Confidence intervals crossing the dotted line indicate *p* > 0.05, and the *x*-axis is presented on the logarithmic scale.

Table 3 and Figure 1 provide the results of logistic regression models for lifetime and past 30-day e-cigarette, cigarette, and dual use. Results indicated multiple significant differences in lifetime and past 30-day e-cigarette, cigarette, and dual use by neighborhood- and individual-level characteristics.

## 4. Discussion

Socioeconomic differences in cigarette smoking among both youth and adults are well established and lead to the development of SES-related health disparities across the life course [3,4,11,13,15]. Rapid increases in e-cigarette use among youth in recent years and the potential for negative health effects from e-cigarette use [5] have sparked substantial concern among health professionals that e-cigarette use will follow similar socioeconomic patterns as combustible cigarettes. If so, e-cigarettes have the potential to exacerbate SES-related health disparities already in place [8]. Results of the current study suggest that, among youth, e-cigarettes may follow a similar socioeconomic pattern compared with combustible cigarettes. Youth living in higher-poverty neighborhoods and youth living in households with lower levels of education were significantly more likely to engage in cigarette, e-cigarette, and dual e-cigarette/cigarette use. Additionally, results of the current study suggest that most youth smoking cigarettes are also using e-cigarettes. As such, any long-term health effects associated with e-cigarette use are likely compounded on top of the known health effects of combustible cigarette use [8]. Socioeconomic differences in dual e-cigarette by neighborhood poverty and household education observed in this study suggest that lower-SES youth are more likely to experience the combined negative health effects of both cigarette and e-cigarette use.

The current study is among the first to examine neighborhood-level differences in e-cigarette use among youth. For those who have investigated this question, Shih and colleagues did not observe neighborhood-level SES differences in e-cigarette use in a community sample of California youth in 2014 and 2015 [16]. It remains possible that e-cigarette use is increasing more rapidly in lower-SES neighborhoods as e-cigarettes become more prevalent [8]. The results of the current study suggest an independent association of neighborhood SES and household SES with youth e-cigarette, cigarette, and dual use after accounting for other individual-level sociodemographic factors in 2019. Similar findings have been echoed by other studies examining both neighborhood- and individual-level risks for youth combustible cigarette use [11,13,24]. Much of this research is based on social ecological theories of human behavior that predict that both distal factors (e.g., neighborhood-level) and proximal factors (e.g., individual-level) have the potential to exert unique influences on the development and maintenance of health behaviors [14]. Additionally, proximal factors are likely to show the strongest associations with behaviors. As expected, based on these theories [14], proximal factors in the current study, such as household SES, were more strongly and consistently associated with youth behavior compared with more distal factors, such as neighborhood SES. Comparison of effect sizes identified household SES as the strongest predictor of youth cigarette, e-cigarette, and dual use. Additionally, household SES maintained significant associations across both lifetime and past 30-day cigarette, e-cigarette, and dual use, further emphasizing the importance of proximal SES for youth health behaviors.

The current study could not directly identify the mechanisms through which neighborhood or household SES may exert influence on youth health behaviors. Previous studies on combustible cigarette use among youth suggest multiple pathways through which neighborhood SES may be associated with youth e-cigarette use. First, lower-SES neighborhoods in the United States tend to house a higher density of tobacco shops and advertising [25,26]. A higher density of tobacco retail locations within lower-SES neighborhoods has been linked to higher youth smoking rates [26]. Given that many traditional tobacco shops have also incorporated sales of e-cigarette devices and liquids in recent years, it is possible that the density of e-cigarette product availability and advertising links lower-SES neighborhoods to increased youth e-cigarette use to some extent. Second, previous studies have suggested that youth living in lower-SES areas may experience more permissive norms related to cigarette smoking and a higher likelihood of encountering smokers in their local areas [11,13]. More permissive smoking norms in lower-SES households are thought to emerge from a combination of reduced access to both health information and preventive services [8]. Cigarette norms and subsequent smoking behaviors are often passed across generations from parents or guardians to children [11,13]. Intergenerational evidence even suggests the presence of links across multiple generations in cigarette smoking [27]. While recent studies have shown that more permissive e-cigarette norms are associated with an increased risk of e-cigarette use among youth [28,29], data on socioeconomic differences in the e-cigarette norms are yet to be presented. Future studies should investigate important questions of the pathways through which neighborhood and household SES are associated with e-cigarette use.

Researchers and public health professionals should continue to monitor e-cigarette, cigarette, and dual use among youth with particular attention paid to potential socioeconomic disparities. Continued surveillance of socioeconomic disparities in youth health behaviors is an important component of identifying and addressing socioeconomic disparities in health outcomes. Research should continue to examine individual- and neighborhood-level socioeconomic disparities in youth e-cigarette, combustible cigarette, and dual use. This information can provide guidance for prevention programs seeking to improve youth health behaviors and reduce socioeconomic disparities in youth health behaviors and outcomes.

Despite the novel contribution of the current study to research on socioeconomic differences in youth health behaviors, the results presented here should be interpreted with caution. First, data are from one state in the United States with strong norms against substance use [29]. It remains possible that states with more permissive norms against substance use may exhibit different associations between socioeconomic factors and e-cigarette, cigarette, and dual use. Second, the cross-sectional analysis presented cannot determine the length of exposure to neighborhood or household SES or the consistency of e-cigarette, cigarette, and dual use. As a result, some youth in this sample may experience limited or no long-term health effects from e-cigarette or cigarette use. Future studies employing longitudinal, representative samples should investigate geographic and temporal differences in youth e-cigarette and cigarette use.

## 5. Conclusions

The current study is among the first to examine neighborhood- and individual-level socioeconomic differences in e-cigarette, cigarette, and dual use among youth. Results suggest that e-cigarette use among youth may follow a similar socioeconomic path as combustible cigarettes [8]. The current study indicated that youth living in higher-poverty neighborhoods and in households with lower levels of education were at an increased risk of e-cigarette, cigarette, and dual use. Additionally, the magnitude of risk was similar across e-cigarette, cigarette, and dual use. Both neighborhood- and individual-level socioeconomic factors were uniquely associated with increased lifetime e-cigarette, cigarette, and dual use, suggesting that SES may simultaneously influence youth behavior at multiple ecological levels [11,14]. Given the recent emergence of the e-cigarette epidemic among youth, early identification of socioeconomic patterns in e-cigarette use is essential to help prevent the compounding of socioeconomic-related health disparities already in place [8].

## Figures and Tables

**Table 1:** Sample characteristics and population estimates.

Variables		*N*	Unweighted M (SD), %	Weighted M (SD), %
Age		85,363	14.0 (2.2)	14.5 (2.9)
Grade				
	6	27,657	32.0%	26.7%
	8	25,581	29.6%	25.4%
	10	20,376	23.6%	24.8%
	12	12,732	14.7%	23.1%
Gender				
	Female	44,382	51.7%	51.1%
	Male	40,776	47.5%	48.5%
	Transgender	299	0.3%	0.2%
	Other	470	0.5%	0.3%
Race/ethnicity				
	AI/AN	3247	3.8%	1.7%
	Asian	2951	3.4%	2.3%
	Black/AA	2357	2.7%	1.8%
	Hispanic/Latino	14,203	16.4%	18.8%
	NH/PI	2284	2.6%	2.0%
	White	69,019	79.9%	75.5%
Highest educated household member				
	High school or less	13,186	18.2%	19.7%
	Some college	10,376	14.3%	14.4%
	College degree	33,168	38.4%	44.6%
	Graduate degree	15,760	21.7%	21.3%
Lifetime e-cigarette use		15,215	18.5%	20.9%
Past 30-day e-cigarette use		7044	8.5%	9.7%
Lifetime cigarette use		5882	7.2%	7.9%
Past 30-day cigarette use		897	1.1%	1.2%
Lifetime dual use		5150	7.2%	8.2%
Past 30-day dual use		723	1.0%	1.1%

Notes. Unweighted *N* = 86,346; M = mean; SD = standard deviation; weighted %’s approximate population characteristics; race/ethnicity categories are not mutually exclusive; American Indian or Alaskan Native (AI/AN), Black or African American (Black/AA), Native Hawaiian or other Pacific Islander (NH/PI).

**Table 2:** Principal component analysis for census-based measures by ZIP code tabulation area.

Variables	NH Poverty	M	SD	Min	Max
Percent of families below the poverty line	0.58	10.63	9.29	0.00	47.56
Percent of individuals receiving public assistance	0.53	15.50	17.22	0.00	100.00
Percent of individuals 25+ without high school diploma	0.49	8.23	7.23	0.00	45.68
Percent of individuals unemployed and in workforce	0.38	4.14	5.50	0.00	50.00
Eigenvalue	2.10				
Percent of variance	52%				

Notes. *N* = 278 zip code tabulation areas (ZCTAs); NH = neighborhood; M = mean; SD = standard deviation; Min = minimum; Max = maximum; 45% of ZCTAs were categorized as rural/small town by RUCA code.

**Table 3:** Results of logistic regression models for neighborhood- and individual-level lifetime and past 30-day e-cigarette use, cigarette use, and dual use.

Variables		Est.	SE	*p*	OR	95% CI	Est.	SE	*p*	OR	95% CI
		**Lifetime e-cigarette use**					**Past 30-day e-cigarette use**				
Neighborhood level											
	NH poverty	0.12	0.03	<0.001	1.12	1.06, 1.19	0.04	0.03	0.212	1.04	0.98, 1.11
	Rural NH	−0.13	0.09	0.127	0.88	0.74, 1.04	−0.17	0.10	0.104	0.84	0.69, 1.04
Individual level											
	HH education	−0.52	0.02	<0.001	0.60	0.57, 0.62	−0.50	0.03	<0.001	0.61	0.57, 0.64
	Grade	0.18	0.04	<0.001	1.20	1.10, 1.30	0.19	0.04	<0.001	1.21	1.11, 1.31
	Age	0.35	0.10	<0.001	1.42	1.17, 1.71	0.24	0.10	0.015	1.27	1.05, 1.53
	Male	0.12	0.02	<0.001	1.12	1.07, 1.18	−0.07	0.03	0.039	0.94	0.88, 0.99
	Non-White	0.42	0.07	<0.001	1.52	1.33, 1.73	0.32	0.08	<0.001	1.38	1.19, 1.60
	Hispanic/Latino	0.15	0.07	0.017	1.17	1.03, 1.32	−0.12	0.08	0.133	0.89	0.77, 1.04
Intercept		−2.50	0.38	<0.001	-	-	−3.40	0.38	<0.001	-	-
		**Lifetime cigarette use**					**Past 30-day cigarette use**				
Neighborhood level											
	NH poverty	0.13	0.03	<0.001	1.14	1.08, 1.21	0.10	0.06	0.083	1.10	0.99, 1.23
	Rural NH	0.09	0.09	0.275	1.10	0.93, 1.30	0.24	0.20	0.226	1.28	0.86, 1.89
Individual level											
	HH education	−0.56	0.03	<0.001	0.57	0.54, 0.61	−0.67	0.08	<0.001	0.51	0.44, 0.59
	Grade	0.12	0.04	0.005	1.13	1.04, 1.23	0.29	0.11	0.007	1.33	1.08, 1.64
	Age	0.32	0.10	0.001	1.37	1.14, 1.66	0.08	0.23	0.738	1.08	0.69, 1.70
	Male	0.17	0.04	<0.001	1.19	1.11, 1.27	0.08	0.11	0.456	1.08	0.88, 1.33
	Non-White	−0.12	0.08	0.108	0.89	0.77, 1.03	−0.62	0.19	0.001	0.54	0.37, 0.79
	Hispanic/Latino	0.34	0.07	<0.001	1.40	1.22, 1.60	0.06	0.15	0.683	1.06	0.79, 1.42
Intercept		−3.14	0.41	<0.001	-	-	−6.30	0.97	<0.001	-	-
		**Lifetime dual use**					**Past 30-day dual use**				
Neighborhood level											
	NH poverty	0.15	0.04	<0.001	1.16	1.08, 1.24	0.08	0.07	0.276	1.08	0.94, 1.23
	Rural NH	0.00	0.11	0.974	1.00	0.81, 1.24	0.08	0.23	0.723	1.08	0.69, 1.70
Individual level											
	HH education	−0.70	0.04	<0.001	0.50	0.46, 0.53	−0.82	0.09	<0.001	0.44	0.37, 0.52
	Grade	0.19	0.05	<0.001	1.21	1.09, 1.34	0.31	0.12	0.009	1.36	1.08, 1.72
	Age	0.36	0.11	0.001	1.44	1.15, 1.79	0.12	0.26	0.649	1.12	0.68, 1.86
	Male	0.17	0.04	<0.001	1.19	1.10, 1.28	0.08	0.12	0.521	1.08	0.86, 1.36
	Non-White	0.14	0.09	0.124	1.15	0.96, 1.38	−0.48	0.21	0.018	0.62	0.41, 0.92
	Hispanic/Latino	0.20	0.08	0.016	1.22	1.04, 1.43	−0.14	0.17	0.409	0.87	0.62, 1.22
Intercept		−3.51	0.49	<0.001	-	-	−6.37	1.08	<0.001	-	-

Notes. *N* = 78,740; ZCTAs = 267; Est. = unstandardized estimate; SE = standard error; *p* = *p*-value; OR = adjusted odds ratio; CI = confidence interval; NH = neighborhood; HH = household; Male, non-White, and Hispanic/Latino were binary variables with 1 coded as the variable name; estimates weighted to reflect population characteristics, stratified by school district, and clustered by ZCTA; age and NH poverty were standardized prior to inclusion in the model.

## Data Availability

Data are available via request from the Utah Department of Health.
